# Comparison of Three Manufacturing Techniques for Sustainable Porous Clay Ceramics

**DOI:** 10.3390/ma14010167

**Published:** 2020-12-31

**Authors:** Fernanda Andreola, Isabella Lancellotti, Rachele Sergi, Valeria Cannillo, Luisa Barbieri

**Affiliations:** 1 Department of Engineering ‘Enzo Ferrari’, University of Modena and Reggio Emilia, Via Vivarelli 10, 41125 Modena, Italy; andreola.fernanda@unimore.it (F.A.); isabella.lancellotti@unimore.it (I.L.); rachele.sergi@unimore.it (R.S.); valeria@unimore.it (V.C.); 2 Interdepartmental Center for Applied Research and Services in Advanced Mechanics and Motoring, INTERMECH-Mo.Re., University of Modena and Reggio Emilia, Via P. Vivarelli 10/1, 41125 Modena, Italy

**Keywords:** porous clay ceramics, manual pelletization, pressing, shell scaffold, fertilizer

## Abstract

This study proposes different manufacturing techniques (manual pelletization, powder pressing, and “shell scaffold”) to obtain lightweight clay ceramics containing recovery raw materials. The sintering in an electrical furnace (1000 °C, 1 h processing time) was conducted by traditional firing from room temperature, for pressed and shell-scaffold samples, while the flash heating (i.e., samples directly put at 1000 °C) was used only for the pellets. The porous materials (porosity 40–80%), functionalized with nutrients (K and P) in amounts to confer the fertilizer capability, gave suitable results in terms of pH (6.7–8.15) and electrical conductivity (0.29–1.33 mS/cm). Thus, such materials can be considered as feasible lightweight clay ceramics, with a positive effect on the soil. These findings permit us to hypothesize a potential use in green roofs or in agronomic applications.

## 1. Introduction

The inspiring principles of sustainability are (i) balanced and lasting economic growth, (ii) social progress and improvement of the quality of life, and (iii) protection and enhancement of the environment.

During the twentieth century, in the world, the use of fossil fuels has grown 12 times, and the extraction of natural resources has grown 34 times [1]. To preserve our planet Earth, help comes from the new circular economy approach. Within it, “the biological and technical nutrients” contribute to maintain the value of products, materials, and resources for the longest possible time, by returning them to the product cycle at the end of their use, thus minimizing the waste generation.

The new circular-economy action plan, in fact, illustrates new initiatives that affect all the product cycles, to modernize and transform our economy, safeguarding the environment. The plan has the ambition to do the following:

- Contribute to the transition towards a regenerative growth model that gives the planet more than it takes, working towards maintaining the consumption of resources within the limits of the planet;
- Double the use of circular materials in the next decade, thus reducing the footprint of European consumption;
- Lead to a GDP growth of 0.5% by 2030, in addition to creating approximately 700,000 new jobs;
- Strengthen the EU industrial base and encourage business creation and entrepreneurship among SMEs.

Since up to 80% of the environmental impact of the products is determined in the design phase, it is essential to transfer from the linear pattern of “take–make–use–dispose” to the circularity of production and the principles of sustainability:

- Longer-lasting, reusable, upgradeable, and repairable products;
- Greater quantity of recycled material in products;
- High-quality remanufacturing and recycling;
- Reduction of the environmental footprint;
- Constraints on disposable products and premature obsolescence;
- Prohibition of destruction of unsold durable goods;
- Promotion of the “product as a service” model;
- Digitization of product information;
- Reward system based on product sustainability.

Therefore, from the point of view of materials, a circular economy should be achieved to improve efficiency in the use of resources and to prevent, or at least reduce, the negative impact linked to the generation and management of waste. Such improvement and innovation could be obtained through the recycling, as well as the reuse, of production and processing waste.

These actions are considered effective to reduce Europe’s dependence on the import of materials and to improve the overall environment and well-being of citizens. The actions and the increased awareness of citizens are driving cultural and economic changes, especially regarding recycled products. Indeed, recycled products are no longer perceived as “class B” assets of lower performance, but rather products of innovation and research, which the economy needs; moreover, they are preferred by most consumers culturally evolved. The European Commission’s circular economy action plan (11 March 2020) establishes the new roadmap until 2022, with strategic and cross-cutting actions to implement the circular economy. From this emerge the following:

1. The primary role of recycling, with expansion of the market for goods with recycled content;
2. Green Public Procurement, the driving factor to increase the demand for sustainable products (mandatory requirement for products with recycled content);
3. Avoid “facade ecology” and admit only certifications, with requirements of independence and objectivity.

The objectives described are pursued through both product and process sustainability, which can be assessed throughout the entire life cycle: raw material acquisition, production, distribution, use, disposal, or recycling (for a product); research and development, design, implementation, operation, dismantling, and site remediation (for a process).

In this research, we focused our attention particularly on some specific aspects: use of residues (in particular of the agro sector, post-consumer, and packaging glass cullet) mixed to a local raw material (red clay, km 0 concept). Such materials were hot-consolidated, comparing three different manufacturing technologies: (i) manual pelletization and flash heating in furnace, (ii) sintering of pressed powders, and (iii) impregnating open-cell natural marine sponges plus traditional firing in furnace. A porous clay ceramic which belongs to clay ceramics materials, a class of materials with a solid market and a significant environmental impact from their manufacture to their distribution, was chosen as the final product. As a matter of fact, some works have been just published by the authors on lightweight ceramic aggregates [2,3,4] which belong to the aggregate materials and represent the second most consumed material after water [5], equal to 2282 million tons in Europe in 2016 [6]. The aggregate family is the main supplier of raw materials for the construction of infrastructures and buildings, as well as for industry and environmental protection, which confers to it a clearly strategic character [5]. Based on the final structure and properties of aggregates, their use has been extended to different applications, ranging from light concrete and green roof to gardening (i.e., substrates in horticulture and hydroponic crops), or others, such as geotechnical applications, sidewalks, or filter media.

Commercial aggregates are produced by drying and sintering of pelletized clay or shale, exploiting the raw materials capability of being “bloated”. This phenomenon is caused by the gas release of the organic or mineral matter contained inside the abovementioned natural raw material during heating. A well-known commercial example is represented by LECA (Light Expanded Clay Aggregate), produced starting from natural clay, containing no harmful substances or organic materials, and certified by the Italian ANAB (National Association for Green Building Architecture)–ICEA (Ethical and Environmental Certification Institute), certification of bio building products according to UNI EN ISO 14,024 [7] type I environmental labeling. By subjecting the clay to an appropriate firing cycle, it swells at 1000–1200 °C due to the action of gases generated within the mass [8,9] that cannot escape from the body (organic components of the clay, water vapor, and steam development). The result of the thermal expansion associated to a vitrification (clinkerization) process at temperatures higher than 1200 °C [10] produces an internal light cellular structure and an external compact and resistant shell, and the whole offers an excellent weight/resistance ratio. The lightness of this product is due to the high percentage (up to 90% of the volume of the particles) of semi-closed porosity.

Most lightweight clay ceramics are obtained by extrusion [11,12] or agglomeration [13,14] by mixing the raw materials with a suitable amount of water in order to obtain a good plasticity for the forming.

In this study, three different manufacturing technologies, two of which are not usually applied for this type of products, were used and compared, namely pressing, pelletization, and “shell scaffold”.

The manufacturing process of lightweight aggregates (LWAs) has the same steps as the structural ceramics, with the substantial difference in the shaping processes, which occur in a pelletizer. Pelletization is a method of agglomeration that permits the particle-size enlargement, in which ceramic powders are processed into pellets or granules of dimensions between 0.5 and 2.0 mm [15]. Pelletization is not a typical forming technique of traditional ceramics; it is used in a multitude of industries (pharmaceutical, food, fertilizer, etc.), to process thousands of materials from difficult-to-handle fine powders into easy-to-handle pellets. A specific advantage of producing LWAs via pelletization is that they are not so dense. Therefore, they are strong enough to hold up to handling and to the specific application, due to the sintering of raw materials during firing, but they can still release nutrients as needed. This is especially valuable when working with fertilizer or soil amendment products [16]. This technique allows many advantages with respect to the manipulation of fine powders: (1) improve handling and application (pellets are easier to feed, due to improved and more consistent flowability) and (2) reduce dust loss [14].

Pressing is a forming technique widely used in the ceramic field, in which granular ceramic materials are made cohesive through mechanical densification. The mechanical features of the sample obtained after cold-forming (i.e., the “green body”) strongly influence the subsequent sintering process and then the mechanical properties of the final piece [17]. Traditional ceramics, such as tiles, are pressed uniaxially, starting from powders with moisture content (5–7 wt.%). The pressures used vary according to the type of product; the more sintered ones (porcelain stoneware) require high pressure (in the order of 40–45 MPa), while for porous products (e.g., monoporosa), the applied pressure drops to about 25 MPa [18]. Uniaxial pressing was applied in the study, as in the most common forming techniques used for traditional ceramics; the chosen pressure was 18 MPa, in order to avoid a too-high compaction level of the powders.

It is worth noting that the third technique was taken from the field of biomaterials and used for the first time in this context. Such an approach is inspired by the concept of translational research and cross-fertilization from other disciplines. Traditionally, scaffolds, i.e., highly porous structures used for bone regeneration, are produced by the classical replica technique, which uses a ceramic slurry to coat a polymeric sponge that is subsequently burned to obtain ceramic foams. A proper thermal treatment permits to burn out the sponge and, at the same time, to make the ceramic powders sinter. Scaffolds obtained by means of this technique show high porosity, which unfortunately results in poor mechanical properties. However, scaffolds should be porous enough and, simultaneously, should have sufficient mechanical strength to be handled. Regrettably, the requirements of porosity and adequate mechanical properties are interlinked and opposed, and this is the reason why the design of scaffolds is very complex but also intriguing. In recent years, an innovative technique for the production of scaffolds has been set up [19,20]. This new approach enabled the delivery of the so called “shell scaffolds”, i.e., more resistant structures, retaining the high porosity of the traditional scaffolds [19,20,21,22,23,24]. In fact, the produced samples were characterized by a compact and permeable surface (the shell), which provides both high permeability to fluids and mechanical support. Such a shell surrounds a highly porous internal network. For these reasons, the new protocol goes beyond the limits of the traditional replica method.

The “shell scaffold” technique was adapted here, for the first time, to produce lightweight clay ceramics as growing media/drainage layers in green roofs or hydroponic crops, by using the red clay and the phosphorus (P) and potassium (K) containing glass (instead of the bioactive glasses used up to now for the production of shell scaffolds for the biomedical field).

The objective of this study was (i) to contribute to the research on porous clay ceramics residues-containing and consolidated by different techniques and (ii) to create prototypes with good weight/resistance ratio, useful as growing media/drainage layers in green roofs or hydroponic crops, in order to generate the following:

(a) Porosity: Spent coffee grounds, as pore forming agents, were mixed together with a ferruginous red clay.
(b) Nutrient effect: Animal bone meal ash and K_2_CO_3_, suppliers of P and K respectively, were added, vitrified by packaging glass cullet.

## 2. Materials and Methods

### 2.1. Raw Materials Used

For the preparation of the specimens, a local ferruginous red clay (Zocca, Modena, Italy) and spent coffee grounds (SCGs) collected by the owner of a bar in Modena (Italy) were used. Due to the high humidity content (65% of water), the SCGs were dried by following a procedure optimized in a previous paper [3]. The use of a pore-forming agent (SCGs) was necessary only in the materials prepared by manual pelletization and pressing, while for those prepared by shell scaffold, due to the use of a sacrificial skeleton that burned during firing, it was not necessary.

The porous clay ceramics were functionalized by phosphorus and potassium, as nutrient elements, added as vitrified by a so-called fertilizer glass (FG). The glass composition was properly designed by the authors, to achieve a controlled release, over time, of P and K [2,25]. A mix of glassy sand (a commercial product obtained by the second treatment of the fraction of packaging glass cullet not destined for glasswork), cattle bone flour ash (CBA) (i.e., phosphorous (P) intake), and potassium carbonate (i.e., potassium (K) intake) was melted at 1450 °C for 2 h. Subsequently, the clay and SCGs were ground and sieved (particle size < 1 mm), while fertilized glass was milled in an agate jar and sieved (particle size < 100 µm). The other raw materials did not need grinding and sieving before use.

All samples, described below, were codified by indicating the manufacturing technique used, i.e., pelletization (PE), pressing (PR), and scaffold shell (SS), followed by (a) REF for the starting matrix of 85 wt.% of clay and 15 wt.% of SCGs or (b) FG for the matrix added, with fertilizer glass indicating also the wt.% of this last (30, 50% with respect to clay plus SCGs). No SCGs were used in the case of shell scaffolds (see the Section 2.4 for details).

### 2.2. Manual Pelletization

At a ceramic mixture of 85 wt.% of clay and 15 wt.% of SCGs, 30 or 50 wt.% (with respect to clay + SCGs) of fertilizer glass was added (see Table 1). The powders were homogenized for 10 min by a slow ball mill and then moistened with proper water content (20–30% wt.%), to obtain the adequate plasticity needed for shaping. Thick cylinders (about 1 cm diameter and about 40 cm long) were manually formed from the well-blended paste; subsequently, they were cut into small cylinders, all of the same size. By hand-working the cylinders, small spheres of the same shape and size were made (1.5–2.0 g of weight and 1–1.5 cm of diameter). To avoid the presence of cracks on the external surface, a surface-smoothing operation was carried out: spraying distilled water on the surface and gently smoothing until the external surface was completely smooth and uniform. Then, samples were dried at 105 ± 5 °C (in a stove), to remove the free water from the body, to avoid cracking that can eventually occur during firing at 1000 °C, which took place in a static furnace (Lenton AWF13/12), as previously reported [2]. Once the set temperature of 1000 °C was reached, the samples (in refractory crucibles) were fired for 1 h. Using this flash-firing process, the samples underwent thermal shock similar to that occurring during the industrial process at around 1300 °C. Then, samples were left to cool at room temperature for 24 h.

### 2.3. Powder Pressing

Two ceramic mixtures were prepared by this technique: the reference, i.e., 85 wt.% of clay and 15 wt.% of SCGs, and the reference added with 30 wt.% of fertilizer glass (Table 1). Each batch composition was prepared homogenizing by slow ball mill for 10 min and then moistened with 7 wt.% of water content. The mixes were maintained in a hermetic plastic bag for 1 h, in order to homogenize the humidity, and again sieved below 2 mm, to obtain suitable press-powder. To obtain cylindrical samples (40 mm diameter and 5 mm thickness) from powders, a hydraulic press (Nannetti model S Mignon, Faenza, Italy) at 18 MPa of pressure was used. Subsequently, samples were sintered by using a static furnace (Lenton AWF13/12): (i) room temperature → 500 °C at 5 °C/min; (ii) 500 °C → 1000 °C at 10 °C/min. The final temperature (1000 °C) was kept constant for 1 h, to allow the total burning of the SCGs and the sintering. Afterward, the samples were allowed to cool down at room temperature.

### 2.4. Scaffolds

The “shell scaffold” technique was adapted to produce samples with red clay and fertilizer glass powder (see Table 1 for compositions). This procedure represents an innovation with respect to the traditional replica method, where (i) the samples are usually squeezed before drying, in order to remove the exceeding slurry, and (ii) the samples are slowly dried. Instead, in the new protocol, the green bodies were kept fully loaded with the slurry during the retrieving and the drying steps.

A natural marine sponge (Spugnificio Rosenfeld, Muggia (TS), Italy), leftover from the industrial production of commercial marine sponges, was employed as a sacrificial template for the scaffolds. In fact, the pore size can be tailored by choosing different skeletons (i.e., sponges) for the process. The clay and fertilizer glass powders were dispersed in distilled water, to obtain the slurry. A polyvinylic binder was added to obtain a slurry with controlled viscosity and to promote the adhesion of powders to the sponge during immersion. Various weight ratios between distilled water, fertilizer glass and clay powders, and binder were examined, to achieve the optimization of slurry and of the technique. After some tests, the slurry was designed according to the following weight composition: 50 wt.% distilled water, 6 wt.% polyvinyl alcohol (PVA), 30.8 wt.% red clay, and 13.2 wt.% of glass.

The slurry was mixed under magnetic stirring. 50 wt.% distilled water and 6 wt.% polyvinyl alcohol were carefully mixed in a beaker, under vigorous stirring until PVA was completely dissolved. Then, 30.8 wt.% of red clay (powders diameter < 1 mm, Argilla Samone 1) and 13.2 wt.% of the fertilizer glass (powders diameter < 100 μm) were carefully added to the distilled water and PVA slurry under stirring and mixed until the obtainment of homogenous slurries. According to Table 1, these samples were named as SS FG30 (the fertilizer glass was 30 wt.% and the clay 70 wt.% of the total powder, to which water and PVA were added)

Moreover, another composition (50 wt.% distilled water, 6 wt.% polyvinyl alcohol, 22 wt.% red clay and 22 wt.% of fertilizer glass) was prepared with the same procedure and named as SS FG50 (50 wt.% FG and 50 wt.% clay).

Subsequently the marine sponges, cut to the desired shape and dimensions (3 × 3 × 5 cm^3^), were manually immersed in the slurry and then extracted from the suspension; as mentioned, unlike the classical replica method, they were not squeezed but kept fully loaded with the slurry. The sponges were immediately dried, as quickly as possible, under a multidirectional vigorous air flux at 100 °C for 10 min. Moreover, the impregnated marine sponges were maintained in rotation into the air flux to keep them slurry-loaded and, at the same time, to guarantee an even distribution of the slurry within the sponges themselves. At the end of the drying process, the sponges resulted to be coated by a thin shell made of clay, glass and binder. Then, the impregnation/drying process was repeated in order to obtain an impregnation as much homogeneous as possible and a thicker outer shell. Finally, a post-forming heat treatment was performed to burn out the organic skeleton (i.e., marine sponge) and densify the network of clay/glass powders.

The samples were introduced into the furnace and were heat-treated, from room temperature to 500 °C, with a heating rate of 3 °C/min, and then subsequently up to 1000 °C at 10 °C/min. The final temperature (1000 °C) was kept constant for 2 h, to allow for the burning of the marine sponge and sintering of the composite structure. Subsequently, the highly porous structures were left to cool down at room temperature.

Additionally, for apparent density measurements and microscopy images, small samples (1 × 1 × 1 cm^3^) were prepared with the same procedure using a cubic sponge.

### 2.5. Samples Characterization

The spent coffee ground, as received, and the powder ceramic mixtures were analyzed by means of Fourier transform infrared (FTIR) analysis, to evaluate the modifications induced in SCGs after firing: (1) 85 wt.% of clay and 15 wt.% of SCGs; (2) 85 wt.% of clay and 15 wt.% of SCGs, added with 30 wt.% of fertilizer glass (with respect to clay + SCGs) after firing. The measure was conducted by using a Spectrophotometer FTIR VERTEX 70 (Leiderdorp, the Netherlands), detector MCT Mid-Band in a range 8000–600 cm^−1^. The analysis was performed by Software OPUS version 5.0.

The chemical analysis of raw materials, fertilizer glass, and waste/by products used was performed by X-ray Fluorescence (XRF), carried out by ARL-ADVANT‘XP+ (Thermo Fisher Scientific Inc., Milano, Italy), software Uniquant.

The physical properties for all the sintered materials were determined as follows: water absorption (WA%) capacity by immersion of samples in distilled water for 24 h. Then, the WA% was calculated by the following equation:

WA% = 100 × (ms − md)/md(1)

where ms is the water-saturated mass after 24 h, and md is the dry mass.

The apparent, ρ_a_, and true, ρ_t_, densities of all sintered samples were evaluated, and the results were used to determine the total porosity (TP):

TP = 100 × (ρ_t_ − ρ_a_)/ρ_t_ (2)

where ρ_a_ was obtained by an envelope density analyzer (GeoPyc 1360, Micromeritics, Wyloway, GA, USA), using a dry medium, while ρ_t_ was determined by a gas (He) pycnometer (AccyPy1330, Micromeritic, Wyloway, GA, USA).

The chemical properties of the materials were assessed by measuring the pH and electrical conductivity. The pH was measured by using a pHmeter (XS Instruments, pH 6, Carpi (MO), Italy), according to UNI-EN 13037 (2012) standard [26] and the electrical specific conductivity (ESC) was measured by an Oakton conductimeter (CON6/TDS6 (OAKTON Instruments P.O. Vernon Hills, IL, USA), according to UNI-EN 13038 (2012) standard [27]. 

The morphological and microstructural characteristics of samples were investigated by scanning electron microscopy (ESEM, QUANTA200 FEI coupled with X-EDS Oxford INCA-350 for chemical analysis, Eindhoven, the Netherland).

Moreover, the permeability of the scaffolds was qualitatively assessed through capillarity tests, in order to evaluate the porosity obtained with the three manufacturing techniques used. A solution with a viscosity similar to that used in green roofs or in agronomic applications (i.e., water plus nutrients) was employed. Green ink was dispersed into the solution, to easily observe the fluid infiltration within the porous material. A face of the sample was put into contact with the solution, and its infiltration driven by capillarity forces was immediately observed.

## 3. Results and Discussion

### 3.1. Raw Materials and Batches Characterization

The red clay chosen, widely used in the ceramic sector, is a ferruginous clay (Fe_2_O_3_ = 7.89 wt.%) with high amount of silica (SiO_2_ = 52.77%), alumina (Al_2_O_3_ = 17.95%), and calcium and magnesium oxides (CaO = 2.57%; MgO = 3.88% wt.%). The loss on ignition (L.o.I) determined at 1050 °C, 2 h, has a value near 10% (humidity, dehydroxylation, and carbonate decomposition). The mineralogical analysis highlighted the presence of the following crystalline phases: quartz [SiO_2_], kaolinitic, muscovitic and chlorite clays, calcium and magnesium carbonate (Dolomite [(Ca, Mg) CO_3_]), and Hematite [Fe_2_O_3_], as chromophore oxide [2]. The thermal behavior of this clay studied by dilatometry technique (not here reported) indicated that the structural consolidation of this kind of clay occurs near 1000 °C after the dehydroxylation at 550 °C. The sintering process causes material contraction, reaching its maximum speed at 900 °C. At 900 °C, reactions between the clay oxides and the calcium oxide formed from the carbonate’s decomposition give rise to phases of neoformation (anorthite-CaAl_2_Si_2_O_8_).

For the pellets and pressed samples, spent coffee grounds (SCGs) were used as porogen agents, due to their high organic content (C = 50.28%), determined by elemental analysis and L.o.I = 98% (1050 °C, 2 h).

The functionalization of the specimens with 15 wt.% of SCGs was performed by adding 30 and 50 wt.% of a fertilizer glass (FG), in order to obtain lightweight materials with the minimum requirements in P and K content for glassy matrix fertilizer: K_2_O ≥ 5%; P_2_O_5_ ≥ 5% and [K_2_O + P_2_O_5_] ~ 10–15 wt.% according to Italian Regulation D.Lgs. 75/2010 art 1 [28]. The fertilizer glass was made by using glassy sand as the matrix and adding cattle bone flour ash and potassium carbonate as sources of P and K, respectively [25]. The chemical composition of the glassy sand expressed in wt.% oxide is as follows: SiO_2_ = 71.70, Al_2_O_3_ = 2.25, Na_2_O = 12.50, K_2_O = 1.00, CaO = 9.50, MgO = 2.00, BaO = 0.04, SrO = 0.37, Fe_2_O_3_ = 0.43, TiO_2_ = 0.07.

The cattle bone flour ash is rich in CaO = 53.49% and P_2_O_5_ = 41.24%, with a low L.o.I = 1.74%, indicating a low unburned carbon content.

Ronga and colleagues [29] outlined the necessity that SCGs are first composted or, in general, subjected to heat treatments at high temperatures before the use in soil, in order to remove the phenolic component of coffee, which is highly phytotoxic and inhibits plant growth.

**Figure 1:**
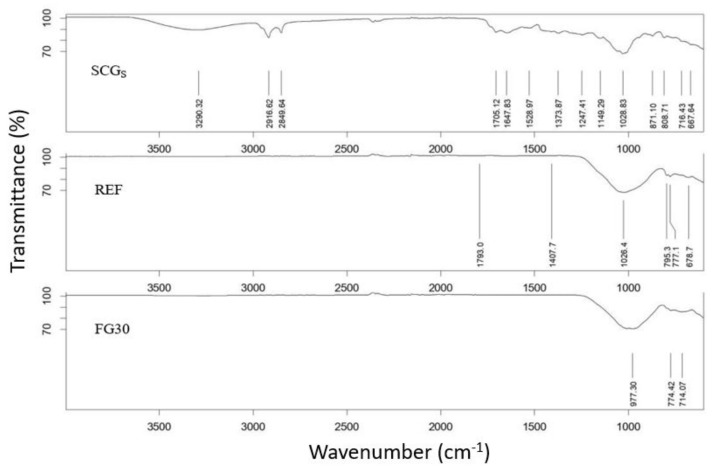
FTIR spectra of SCGs as received (SCGs); matrix composition without fertilizer glass, and matrix composition with 30 wt.% of fertilizer glass.

With the aim of demonstrating that after firing the pore-forming agent used in pellets and pressed samples disappears, in Figure 1, the IR spectra of the SCGs as received and the compositions (clay + SCGs) prepared with and without 30 wt.% of FG are reported.

For the SCGs as received, according to other authors [30], the spectra indicated the broad band observed at 3290 cm^−1^ corresponds to the stretching of O–H group due to inter- and intra-molecular hydrogen bonding of polymeric compounds, such as alcohols, phenols, and carboxylic acid. The O–H stretching vibrations occur within a broad range of frequencies indicating the presence of free hydroxyl groups and bonded O–H bands of carboxylic acids. The presence of caffeine is confirmed by characteristic peaks of C–H stretching (2850–3000 cm^−1^), which correspond to the presence of 3-methyl groups. Besides this, the C=O stretching carboxyl linkage resulting from xanthine derivatives such as caffeine was identified around 1700 cm^−1^.

By comparing this spectrum with those corresponding to the ceramic compositions with and without 30 wt.% fertilized glass after firing, it is possible to note that the characteristic peaks derived from compounds of SCGs disappeared. Only a strong peak around 1000 cm^−1^ is observed, probably derived from the presence of Si–O–Si bond relative to the silicates compounds formed after firing in the reference matrix and belonging to the glass network in the composition containing fertilizer glass.

These findings permit us to confirm the role of spent coffee grounds into the compositions: Due to its organic substances content, it acts as pore forming agent, and during the combustion, it brings an energetic support in the firing step, confirmed by the high calorific power value (4046 Kcal/kg). After firing, the organic compounds disappeared, avoiding any pollution into the soil.

It is known that the expansion behavior on heating of clayed materials depends on different factors: chemical composition of raw materials; amount and viscosity of liquid phase; gasses emission with development of bubbles; and bloating capacity [31]. In the literature, there are several studies that permit us to predict the “bloating” and “non-bloating” features of the materials from the chemical composition of the batch, by locating the mixtures within a specific area in a ternary diagram [32,33]. It is worth noting that the waste materials can contain significant amounts of elements usually not considered (because they are present in trace amounts) but important for the definition of the abovementioned behavior. These components are P_2_O_5_, B_2_O_3_, and heavy metals (Ba, Cr, Mn, Pb, Sr, and Zn), e.g., phosphorus is present in relevant quantities in sewage sludges [2,34,35], cattle bone flour ash [25], and bagasse from beer production [2,36]. The technological behavior of porous clay ceramic batches is remarkably affected by P_2_O_5_, B_2_O_3_, and heavy metals. Thus, the original Riley’s and Cougny’s parameters were adjusted to consider these additional components. Taking into account these modifications in our study, we applied the Cougny modified scheme [33]; it considers the presence of Al_2_O_3_, Fe_2_O_3_, and fluxes (alkali and alkali earth oxides) added with B_2_O_3_ and P_2_O_5_:

(Al_2_O_3_ + TiO_2_) − (Fe_2_O_3_ + CrO_2_ + MnO + PbO + ZnO) − (MgO + CaO + Na_2_O + K_2_O + SrO + BaO + P_2_O_5_ + B_2_O_3_)

**Figure 2:**
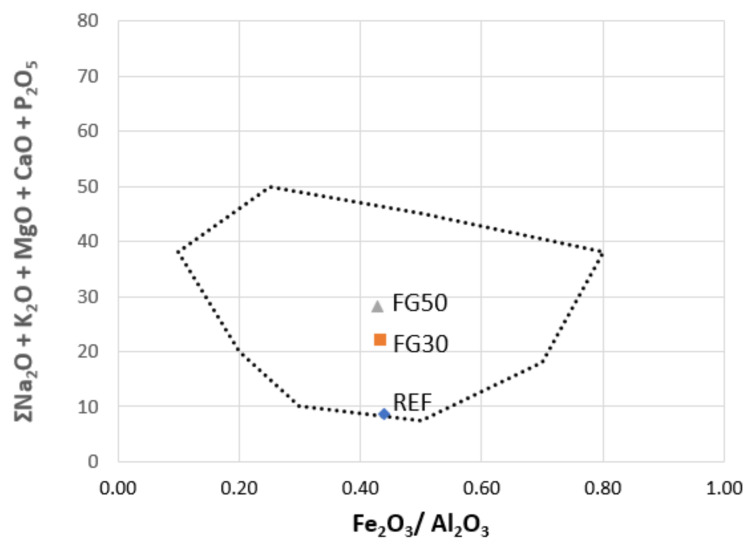
Location of REF, FG30, and FG50 compositions in the Cougny modified predictive diagram.

In the simplified version by Dondi et al. [31], the ternary diagram was redrawn into a binary one, by merging two of the three parameters. Figure 2 shows the localization of raw ceramics’ compositions, highlighting the bloating area with in dotted line. If the different mixtures are located into the bloating area, it is expected that they produce an enough viscous phase able to trap a significant amount of gas and the proper chemical features to expand during thermal treatment. It is possible to observe that the addition of fertilizer glass, with consequent increase in the fluxes content, causes the compositions FG30 and FG50 to be more in the center of the swelling zone than the reference one. The position in the diagram allows us to predict the formation of lightened medium density ceramics. As referred by Dondi et al., for a complete prediction of the bloating behavior, it is necessary to consider other factors that influence the thermal performance of the mixtures: particle and pellet size, viscosity and composition of the glassy phase, maximum temperature and cycle firing, characteristics of kiln and atmosphere, and bloating agents [31].

### 3.2. Samples’ Characterization

To investigate the silica porous materials, water absorption, absolute (true) and bulk (apparent) densities, and total porosity (Table 2), capillarity tests and microstructural morphologies were investigated as a function of the manufacturing techniques.

Regarding a possible application of these materials in the agricultural sector or green roofs, specific physicochemical characteristics need to belong to a suitable range of values, such as porosity >40%, bulk density between 500.00 and 1200.00 kg/m^3^, pH value in the range of 6.5–7.5, and electrical conductivity (EC) < 2.00 mS/cm, [36,37]. The pH and electrical conductibility were considered as soil parameters to confirm their counting of inert materials suitable as a growth media.

Physical (Table 2) and chemical (Table 3) properties of pellets samples [3] were compared with those obtained by pressing and shell scaffold.

The apparent density values show that a significant increase is related to the composition having glass with respect to the reference ones, independently of the manufacturing technique used. This result indicates that the addition of glass (necessary for the nutrients supply) in the mixture improves the sintering, reducing the porosity. Indeed, when comparing porosity data of mixtures with or without fertilizer glass, we see that the values agree with the apparent density ones and confirm the fluxing role of glass in the clay mixture. The most porous samples are those produced by the SS technique, as expected. Water absorption is correlated to the open porosity and shows a similar trend.

Regarding chemical properties (Table 3), pellets showed the best pH results, with values falling in the neutral condition, more suitable for compounds that should act as fertilizers, avoiding high pH variations of the soil. Pressed and scaffold samples containing fertilizer glass highlighted values slightly higher in respect to neutral conditions. Concerning conductivity (E.C.), all porous materials manufactured fell within the limit generally applied for fertilizer compounds (<2 mS/cm).

The thermal behavior of the materials involved in the mixtures was investigated by the TG/DTA techniques [2]. The main events related to clay are as follows: combustion of organic compounds, (around 250 °C, exothermic); endothermic peaks of loss of moisture, around 100 °C; dehydroxylation of water chemically bound around 550 °C; carbonates decomposition, at about 800 °C; and fusion around 1200 °C. The associated weight losses (%) are humidity (1.89 wt.%), dehydroxylation (4.40 wt.%), and carbonates decomposition (4.74 wt.%). For spent coffee grounds, several exothermic peaks were detectable in the range from 250 to 600 °C; the main one is at 300 °C, due to the combustion of the organic fraction (weight loss near to 50 wt.%). The employment of 1000 °C as firing T allows both clay sintering and organic fraction degradation, with corresponding pore formation within LWAs.

**Figure 3:**
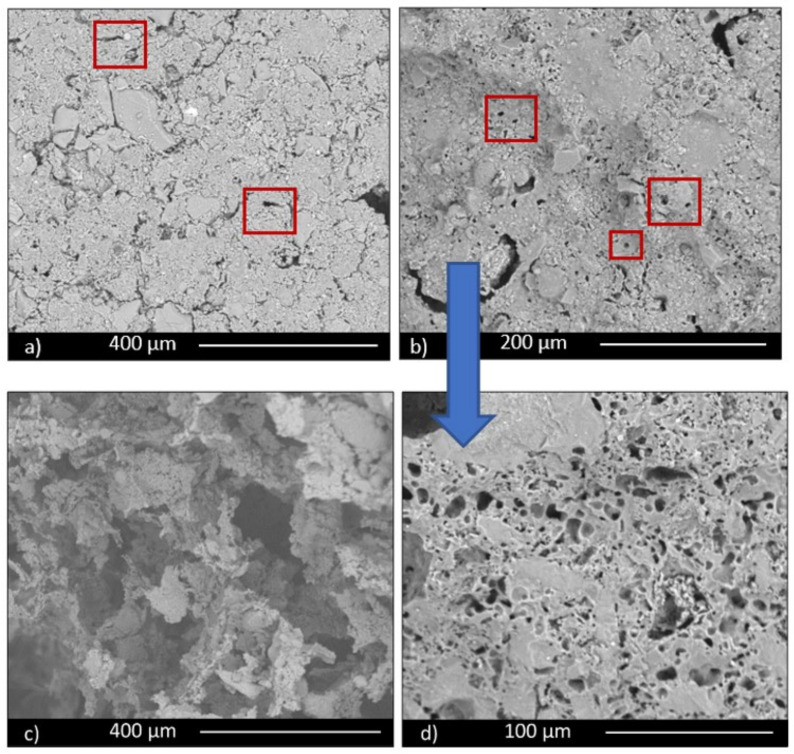
Micrographs of samples: (**a**) PR.FG30, obtained by pressing; (**b**) PE.FG30, obtained by pelletization; (**c**) scaffolds (SS FG30); (**d**) higher magnification image of sample (**b**).

Microstructural characterization was performed by electron microscopy. All three series of samples showed a porous structure, but with significant differences among them. A comparison of the three manufacturing techniques is shown in Figure 3 for the specimens containing 30 wt.% FG, from which it is evident that the higher porosity corresponds to scaffolds; it is more similar for the other two techniques, but slightly lower for pressing technique, notwithstanding the low pressure used to obtain the samples. An intermediate value is observable for pelletization. For these last samples, small spherical pores are evident in Figure 3b (highlighted with red rectangles) and in its magnification (Figure 3d), while for pressed samples, only intergranular irregular pores are shown (Figure 3a, highlighted with red rectangles as well). These data confirm the porosity values, which correspond to 39%, 45%, and 83%, respectively, for pressing, pelletization, and scaffold techniques.

**Figure 4:**
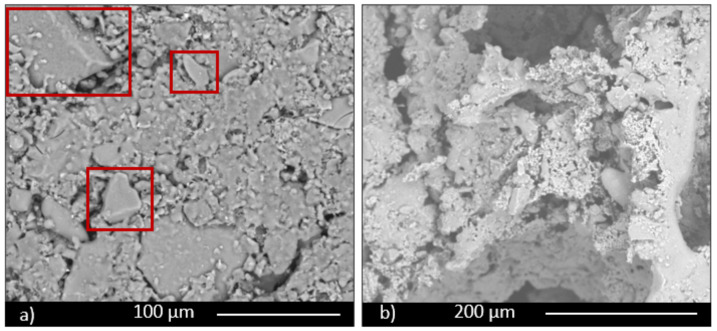
SEM micrographs: (**a**) PR.FG30 and (**b**) scaffold (SS FG30) samples, showing undissolved glass particles.

In all the samples it is possible to observe glass grains undissolved in the microstructure due to the low firing temperature, not enough to completely melt the glass. Particles are particularly evident in samples obtained by pressing (Figure 4a, red rectangles).

**Figure 5:**
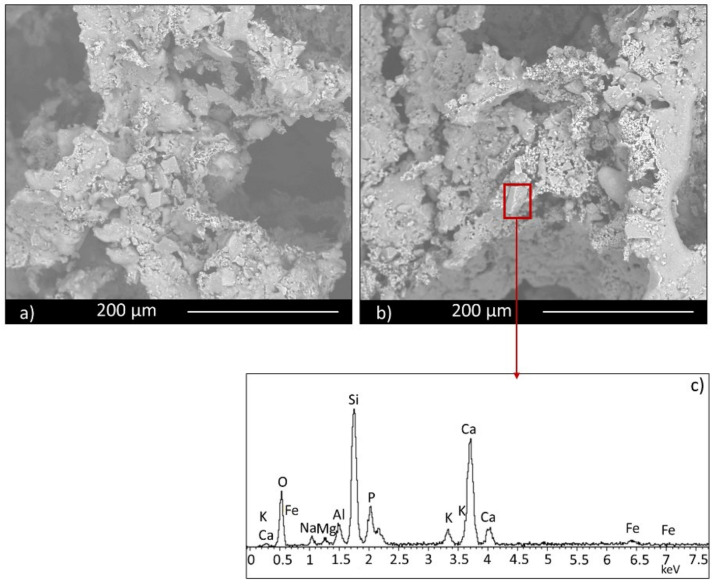
Scaffold (SS FG30) surface (**a**), section (**b**), and EDS analysis (**c**).

**Figure 6:**
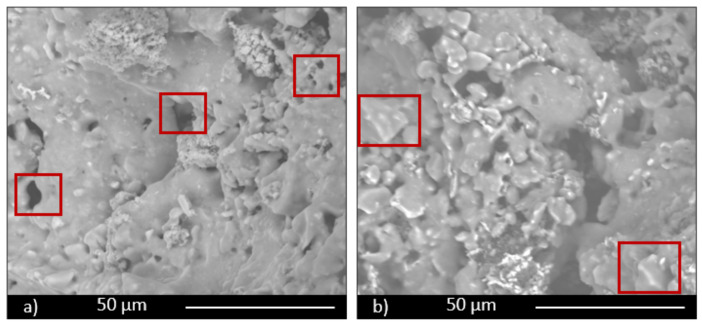
Magnification of images of scaffold (SS FG30) surface (**a**) and section (**b**).

The scaffold sample containing 30 wt.% of glass (SS FG30, Figure 5 and Figure 6) shows high interconnected and irregular porosity (Figure 6a, red rectangles), both on the surface and in the section with pores with a dimension of hundreds of microns. Fertilizer glass particles are evidently dispersed in the materials, with particles dimension less than 100 μm. EDS analysis confirms the glass presence, showing a chemical composition mainly rich in Si, Ca, P, Al, and K. In particular, the nutrient element P presents a percentage in the range of 5–5.5 wt.% both in samples containing 30 and 50 wt.% of fertilizer glass. On the contrary, the nutrient element K presents a percentage in the range of 1–10 wt.% in samples containing 30 and 50 wt.% of fertilizer glass.

Glass particles show squared shapes (Figure 6b, red rectangles). The presence of glass provokes inside the material melted areas due to partial glass melting during the firing process.

**Figure 7:**
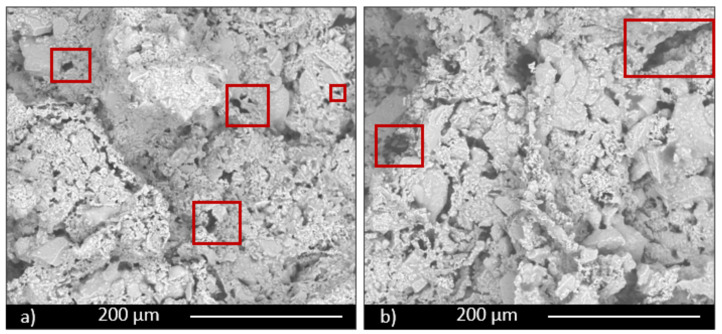
Scaffold (SS FG50) surface (**a**) and section (**b**).

**Figure 8:**
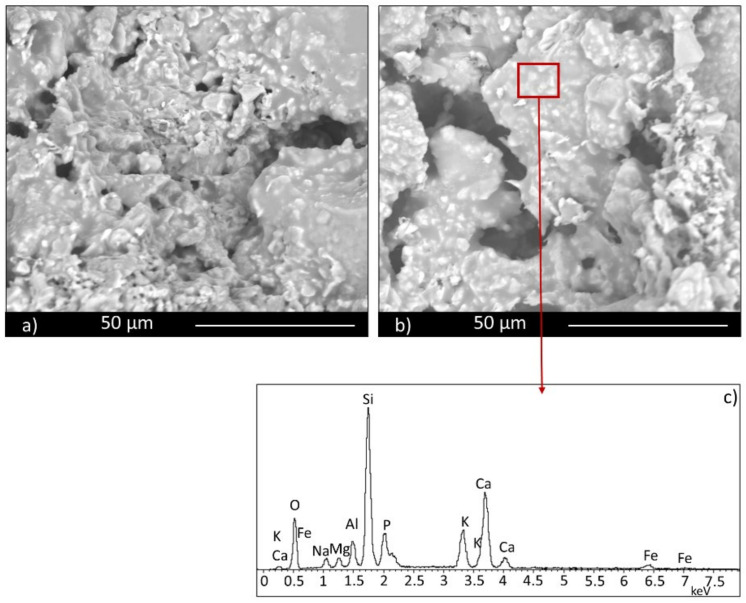
Scaffold (SS FG50) surface (**a**), section (**b**), and EDS analysis (**c**).

The sample containing 50 wt.% of FG (SS FG50, Figure 7 and Figure 8) shows high porosity, as well, but a different microstructure in terms of porosity dimension. Some large pores, but mainly small pores with irregular shape, are evident, particularly along the grain boundaries both on the surface and section of the sample (Figure 7a,b, red rectangles). For this composition as well, glass particles show a high content of the nutrient elements P and K, as well as all the characteristic components of glass, such as Si and Ca. Partially melted areas are also evident, due to the liquid phase formed by the glass during firing and responsible for the slight decrease in total porosity of this composition.

On the other hand, the determination of permeability is of pivotal importance to guarantee adequate nourishment to the plants. Permeability strongly correlates with the presence of a highly interconnected pore network. In fact, the produced scaffolds have an original structure with dissimilarities between the exterior and the interior parts. Such scaffolds, as evidenced from the SEM images, showed an inner high porosity with interconnected pores of different size and an outer surface, which is more resistant and easy to handle. Such an interconnected porosity should be sufficient to ensure an excellent permeability. The other samples are less porous, as also confirmed by the data in Table 1 and SEM analysis.

**Figure 9:**
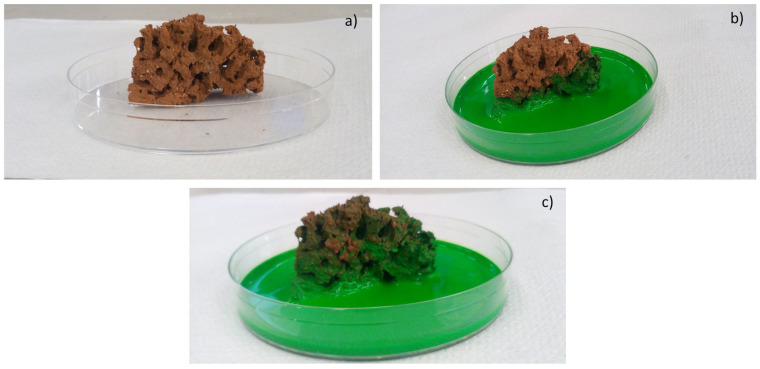
Images of the scaffold (SS FG50) before the contact with green ink (**a**); 2 s (**b**) and 2 min (**c**) after the contact with green ink.

Figure 9 reports the outcomes of the capillarity tests performed on the produced samples in order to qualitatively investigate their permeability. The samples before and immediately after the test are reported. Figure 9a shows the scaffold before the contact with green ink; Figure 9b shows the scaffold 2 s after contact with green ink, and Figure 9c shows the scaffold 2 min after the contact with green ink. In about 2 s, the scaffold started to become green as a result of the fluid infiltration through the pore network. After 2 min, the green ink almost reached homogeneously the upper part of the scaffold, indicating a good capillarity, even though the size and dimension of pores are not regular. The scaffolds were uniformly impregnated, and the rapid infiltration of green ink from the bottom to the upper part of scaffolds confirmed the permeability of the structures. The degree of interconnection of the porous structure, the absence of clogged pores, and the permeability are key features for successful applications. The good permeability of scaffolds could improve physical properties of the ground, reducing the cohesion of clay and ground and counteracting phenomena of erosion. Furthermore, such capillarity can ameliorate the release of nutrients for plants when water goes through the structure of scaffolds. In fact, the fertilizer glass could release the principal nutrients for plants, such as phosphorus and potassium. In this regard, by tailoring the composition of the fertilizer glass, it would be possible to release different ions, like calcium, magnesium, sulfur, copper, and iron, which represent important nutrients for plants. It is worth noting that the time and concentration of ions released influence the growth of plants. Therefore, a controlled and slow release of nutrients in the ground, from inorganic composite materials, could represent an important goal.

**Figure 10:**
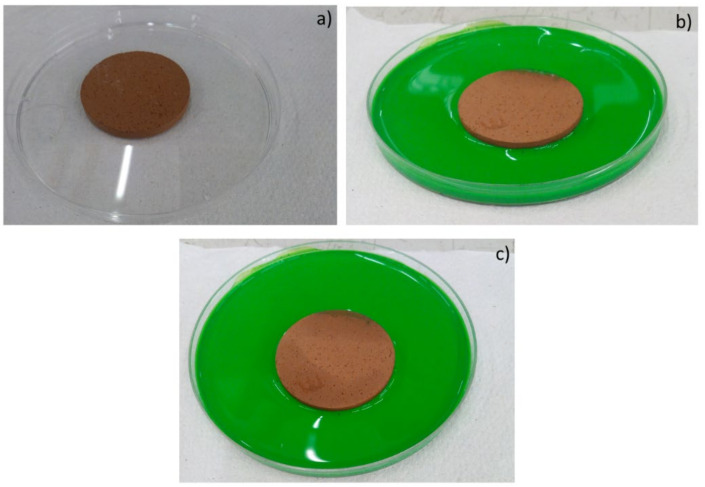
Images of powder-pressed samples (PR.FG30) before the contact with green ink (**a**); 2 s (**b**) and 2 min (**c**) after the contact with green ink.

**Figure 11:**
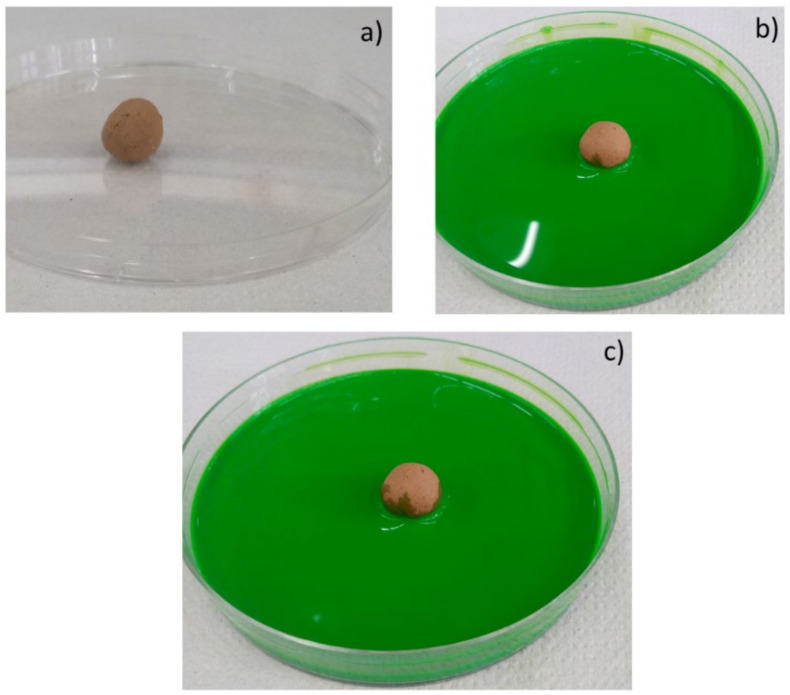
Images of manual pelletized samples (PE.FG30) before the contact with green ink (**a**); 2 s (**b**) and 2 min (**c**) after the contact with green ink.

**Figure 12:**
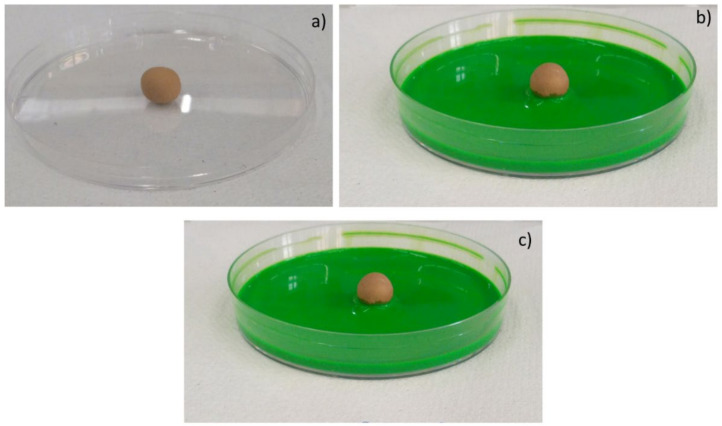
Images of manual pelletized samples (PE.FG50) before the contact with green ink (**a**); 2 s (**b**) and 2 min (**c**) after the contact with green ink.

The same tests were also repeated for the samples produced by pelletization and powder-pressing. As it can be seen in Figure 10, Figure 11 and Figure 12, such samples resulted in being less permeable than the shell scaffolds. Such results are consistent also with the porosity values calculated in Table 2.

## 4. Conclusions

Three different techniques, namely manual pelletization, powder-pressing, and shell scaffold, were effectively employed to produce sustainable porous clay ceramics. All the manufacturing techniques resulted in being suitable for preparing the samples and thus can be considered feasible routes for the production of residue-based porous ceramics. The physical and chemical properties (such as density, porosity, pH, and electrical conductivity) resulted in being acceptable, with respect to the intended use, as per current regulations. In Table 4, schematic summary of all the measured properties is proposed.

In details, comparing the different manufacturing techniques, the powder-pressing resulted in being the less suitable for obtaining lightweight materials, as expected, even if the applied pressure was low. On the other hand, pelletization permitted to obtain lightweight materials with good resistance, in order to support handling, storage, and transportation. It is expected that, while inserted into the soil, such materials can also break, to allow the release of nutrients.

Finally, the samples prepared by scaffold-shell technique showed the higher porosity; however, such a feature goes hand in hand with less resistance.

Thus, depending on the specific use, one technique can be preferable as compared to the others.

Future developments of this research will also explore other aspects, such as mechanical properties of the products, particularly the new shell scaffolds.

## Figures and Tables

**Table 1:** Silica porous samples obtained by the different techniques.

Sample Name	Technique	Fertilizer Glass (wt.%) with Respect to (Clay + SCGs *)
PE.REF	Pelletization	0
PE.FG30	Pelletization	30
PE.FG50	Pelletization	50
PR.REF	Powder pressing	0
PR.FG30	Powder pressing	30
SS FG30	Shell scaffold	30
SS FG50	Shell scaffold	50

*: No spent coffee grounds (SCGs) were used for shell scaffolds.

**Table 2:** Physical properties of the silica porous materials obtained by the different techniques.

Sample Name	WA%	Apparent Density (kg/m^3^)	True Density (kg/m^3^)	Total Porosity (TP%)
PE.REF	17.14	1237	2781	55.51
PE.FG30	14.29	1400	2570	45.35
PE.FG50	12.04	1400	2781	43.63
PR.REF	17.30	1463	2721	46.20
PR.FG30	12.57	1664	2729	39.03
SS FG30	56.27	449	2694	83.33
SS FG50	53.60	458	2683	82.93

**Table 3:** Chemical properties of the silica porous materials obtained by the different techniques.

Sample Name	pH	E.C.* (mS/cm)
PE.REF	6.80	0.40
PE.FG30	6.96	0.29
PE.FG50	6.75	0.36
PR.REF	6.70	1.33
PR.FG30	7.90	0.62
SS FG30	6.80	1.11
SS FG50	8.15	0.46

*: Electrical conductivity.

**Table 4:** Schematic comparison of the measured properties for the three studied techniques.

Measured Properties	Pressing	Pelletization	Scaffold
Water absorption	+	+	+++
Apparent density	+	++	+++
Porosity	+	++	+++
pH	++	+++	++
Electrical conductivity	+++	+++	+++
Capillarity	+	++	+++

+: quite good; ++: good; and +++: very good/excellent.

## Data Availability

Data is contained within the article.
